# Medical Items and News of Interest to the Physician

**Published:** 1871-01

**Authors:** 


### Medical Items and News,

### Of Interest to the Profession.

CULLED FROM THE STANDARD MEDICAL JOURNALS OF THE WORLD.

NOTE.—These formulæ are intended only for the regular practitioner of medicine, and should be employed only by him, or under his immediate supervision.

**Phthisical Sweating.**

M. J. Guyot has found the phosphate of lime, in two to six gramme doses a day, to be a capital remedy for the sweating of phthisis and rheumatism.—*Med. Record.*

**Treatment of Hysteria.**

M. Guillemin, in *Lyon Medical*, recommends the inhalation of etherial tict. of valerian, for the space of one or two minutes, as most successful in cutting short an hysterical seizure.

**Chronic Dysentery.**

Dr. W. E. Whitehead, in the *Pacific Med. & Surg. Journal*, recommends large doses of ipecac (grs. viij—xv ter die) in the treatment of chronic dysentery.

**Erysipelas.**

As an application, the Tincture ferri chloridi is far superior to the tincture of iodine. We have noticed many articles in the different medical journals, recently, attesting the value of this remedy as a local application in erysipelas.

**To Detect Lung Tissue.**

Liquefy the sputum with pure caustic soda, when any particles of lung tissue which may be contained in it will fall to the bottom of the vessel, where it can be secured and placed under the microscope.

**Cazenave's Depilatory Ointment.**

The subjoined safe depilatory ointment is recommended by the French surgeon, Cazenave: Quicklime, one part; Carbonate of soda, two parts; Lard, eight parts. Mix to form an ointment.—*Medical Record.*

**Puerperal Convulsions.**

Dr. Bonticon, of Troy N. Y. writes to the *N. Y. Medical Journal* that he recently adminstered morphia hypodermically, to several patients, suffering from puerperal convulsions, with the happiest results. The convulsions were immediately controlled by the subcutaneous injection of morphia.

**Paris Medical Students.**

All medical students in Paris, who complete their third year of study are invited to become Assistant Surgeons in the military hospitals. More than one hundred have answered to the call and received their appointments.

**Chlorate of Potash in Gonorrhœa.**

Pascual Cardialy Sanchez recomends (St. Louis *Med. & Surg. Jour.*), injections of chlorate of potash—16 grs. to the ounce—in the second stage of gonorrhœa and gleet. The injection should be frequently repeated and continued several days after the discharge has ceased.

**Intermittent Fever.**

The *Pacific Medical & Surgical Journal* says "on the authority of Calvert & Co., of Manchester, it is stated that carbolic acid is a valuable remedy in intermittent fever, in doses of two-thirds of a grain in twenty minims of water, administered hypodermically."

**Iodoform.**

Dr. S. Kennedy, of Newark, Del., (*Med. and Surg. Reporter,*) recommends iodoform as an addition to the ordinary plasters and ointments for syphilitic periostitis. An ointment containing from 30 to 60 grains of iodoform to an ounce of lard is a beneficial remedy to painful burns, sores, chancres, and boils, promoting rapid healing. In two cases of chancre the dry powder was applied with magical results.

**To Preserve Anatomical Specimens.**

Dr. C. Mihn, Pharmacist to Necker Hospital, Paris, (*Bull. Gen. de Therap.*), recommends the subjoined formula as a preserving fluid :—

| | | |
|---|---|---|
| Arsenious Acid | 20 | parts. |
| Crystallized Carbolic Acid | 10 | " |
| Alcohol | 300 | " |
| Distilled Water | 700 | " |

**Chronic Indigestion.**

Dr. Chambers' favorite tonic in this affliction is quinine, in two-grain doses in lemon juice sufficient to dissolve it, and diluted with water to a convenient bulk. Its action seems to be principally on the mucous membrane of the mouth œsophagus, and stomach, which it astringes and tones up to a healthy state, restraining the secretion of mucus, and making the special secretions more active. To quinine he usually adds from 1-24 to 1-20 of a grain of hydrochlorate of strychnia, unless there are some contra-indications to its use.—*Med. Record.*

**Important Visitors.**

It is announced that Prof. Tyndall, the physicist, Dr. Hooker, the renowned English Botanist, and Prof. Huxley, the Zoôlogist, will visit the United States and Canada in 1872.

**Vomiting in Pregnancy.**

Dr. Carl Both, (*Boston Jour. of the Gynæc. Soc.*) highly recommends, in this troublesome complaint,raw meat chopped up very fine and slightly salted. The dose should be small at first, and then gradually increased. [We suppose beef is meant. Ed.]

**Digestion of Cod Liver Oil.**

The *Chicago Med. Times* says that stomachs that resist cod liver oil, or fail to digest it, are made to yield by administering the subnitrate of bismuth, in 10 grain doses, four times daily, in connection with the oil.

**After Pains.**

Dr. J. B. Changnon, in the *Canada Med. Journal*, recommends citric acid for the pains following labor, and declares that it has never failed in his hands. He gives five grains in two or three ounces of water, every five hours. It acts as a nervine, and as a preventive of inflammation.

**Softening of Brain.**

Dr. Forbes Winslow (*Obscure Diseases of the Brain*,) publishes instances in which circumscribed conditions of impaired sensation were the premonitory symptoms of softening of the brain; the defective feeling being manifested in some cases in the skin, in the tongue and fauces.

**Uterine Hemorrhage.**

Prof. Beneke, of Marburg, (*Med. Mirror*), has arrested hemorrhage from the uterus, in several cases, by the application of heat to the spine. He thinks much can and will be done by the principle of applying cold and heat to the spine. So far as the heat is concerned, his experience has fully convinced him of its extreme usefulness in certain cases.

**Glandular Enlargement.**

Jos. Adolphus, M. D., of Des Moines, Iowa, (*Chicago Medical Times*,) recommends this formula in cases of obstinate glandular enlargements:–

℞.
Ipecac., ℥ ss;
Podophyllin, gr. v;
Quinine, gr. xv.

Make into 30 pills. One three times daily.

**Malignant Pustule.**

Dr. Casper, of Stassfurth, (*N. Y. Med. Jour.*, asserts that he has treated several hundred cases of malignant pustule successfully by strong solution of ammonia, and that all the patients, but one, recovered. The dose for a child is one, two, or three drops, and for adults four drops, given every hour, day and night, with sweetened water. The treatment must be continued until the inflammation ceases around the pustule.

**Asthma.**

The Spanish physicians are in the habit of employing arsenite of antimony in asthma. It is given in pills combined with muriate of morphia The dose is two milligrammes from three to five times a day. It is believed by the profession in Spain that arsenic not only acts through the nervous system, but has also a special influence on the walls of the pulmonany air-cells, restoring to them their elasticity.

**Colorless Tincture of Iodine.**

The application of tincture of iodine is often objected to by reason of its coloring properties. It can be rendered perfectly colorless and at the same time retain all its active properties, in the following manner:—

Take:—
Tincture iodine, ʒj;
Water of Amonia, concent., ℥ ij;
Carbolic acid—"Calvert's," gtt. viij. M.

**Ovarian Neuralgia.**

Dr. J. S. Newman reports three cases of this affliction (in the *Chicago Med. Examiner*,) in which the symptoms were similar. For some time they had painful menstruation, considerable fullness in the iliac region, with great tenderness and severe pains shooting down the thighs to knees, and up to base of brain. Upon the adminstration of muriate of amonia and tr. of aconite the trouble disappeared in a few days.

**Nævus.**

Mr. Bateman in the *Lancet*, calls attention to the tartar-emetic plaster for the cure of this sort of tumors, he having used it successfully for fifteen years. His formula is as follows:—

Take:—

Ant. of pot. tart., 1 part;

Ung. resinæ. 2 parts.

Mix and spread on thin linen or leather.

When the skin is not easily blistered, or the nævus is thick, equal parts of the two ingredients must be used. The pustulation must be repeated again and again until the tumor sloughs. By this means one case—a child with 25 nævi on various parts of the head, trunk and extremities—was cured.

---

**Epilepsy of the Retina.**

Dr. Hulings Jackson (*Royal Ophthalmic Hosp. Report*) has pointed out a peculiar condition of the retina, which he noticed in a patient with epileptiform convulsions, and which he calls *epilepsy of retina.* The retina is entirely anæmic, a condition dependent, in all probability, upon a contraction of the retinal vessels, similar to that which occurs in the vessels of the brain during an epileptic fit.

---

**Oxaluria.**

Dr. H. S. Thorne, of Chicago, *Mich. Univ. Med. Jour.*, gave the subjoined prescription to a man æt. 45, afflicted with dyspepsia and oxaluria: Per manganate of potassa, in grain doses, to be made into pills with bread, three to be taken per day, for ten days. He, few days after returned and stated that he was cured. On examination of his urine not a crystal of oxalate of lime could be seen. The symptoms, including the dyspepsia, disappeared.

---

**Prevention of Scarlet Fever.**

Dr. Searcy believes (*Trans. Tenn. Med. Soc.*) that the tonsils are the medium through which the system is contaminated by scarlet fever. He contends that he has abated attacks or mitigated the violence of the disease, by swabbing the throat twice daily, for several days, with a solution of nit. argenti, in the proportion of one drachm to the ounce of water. This must be done the moment the first symptoms of the disease make their appearance.

---

**Coughs.**

For the harrassing and annoying coughs that frequently accompany many accute diseases, and arising from nervous irritation of the larynx, pharynx, palate, or other parts of the throat, an invaluable remedy is:

℞.

Sulph. morphia, gr. j;

Dil. Sul. acid, ʒ j;

Simple syrup, ℥ ij. M.

Half a teaspoonful to be given upon the tongue, and swallowed slowly. The persistent hackings of bronchial difficulties, and even of consumption, are often speedily relieved by it.—*Boston Jour. Chem.*

**Post Partum and Secondary Hemorrhage.**

Dr. Hall Davis (*British Med. Journal*), reports three successful cases with the following treatment:—After all other measures had failed, he cleared out the clots, and injected into the uterus a solution of the perchloride of iron,—one part to four of water. In one case, which had hemorrhage twelve days after delivery, the os uteri was dilated with sponge tents and Barnes' dilators, before the injection was used. He has found the persulphate of iron of equal efficacy.

---

**To Render Hydrate of Chloral Palatable.**

The following combination serves to intensify the action of the chloral, and covers the acrid taste:—

℞.

Hydrate of Chloral, ʒ ss;

Chloroform Water, ʒ ij;

Syrup orange, or tolu, ʒ iij;

Tincture of ginger, gtt. viij;

Water, f. ℥ jss. M.

The Chloroform water is prepared by dissolving half a fluid once of chloroform in one gallon of water.

---

**Cholera Infantum.**

A physician, in the *Am. Pract.*, recommends the following formula, when vomiting and purging are prominent symptoms, in cholera infantum:

℞.

Tinct. opii, gtt. xvi;

Pot. bicarb. ʒ. ss;

Syrp. simplicis, ʒ. iij.

Aquæ menth. pip. ʒ. x. M.

Sig. Administer one teaspoonful after each act of vomiting or purging.

---

**Temporary Blindness following Intermittent Fever.**

Dr. Dutzman (*Wiener Medical Presse,*) reports that a robust young man had been suffering with intermittens tertiana for a fortnight, when a new and more severe attack, complicated with violent chronic convulsions and entire insensibility, reduced the power of vision to feeble perception of light. On ophthalmoscopic examination, nothing abnormal was discovered in the fundus of the eye. The patient took a strong dose of quinine, remained five hours in the same condition, then fell asleep, and when he awoke his sight was completely restored. Pigmentary embolism of the central retinal artery, with consequent transitory disturbance of vision, is suggested as a possible cause.

**Neuralgic Pill.**

Dr. T. C. Osborn of Greensboro, Ala., writes to the *New Orleans Jour. of Med.* that he has used the following pill, with much success in neuralgia :—

℞.

Zinci cyanuretum, gr. vj;
Quiniæ sulphas, gr. ix;
Morphiæ sulphas. gr. iss;
Ext. Belladonnæ, gr. iij.
Fiat pilulæ, no. vj.

S. One pill every 6 hours until the pain is relieved.

**Gargle in Scarlet Fever.**

The *Chicago Med. Exam.*, recommends the following:—

℞.

Alcohol, ℥ss;

Elix. of vitrol. ʒ ij;

Sul. Alum, ʒ j;

Creasote, gtt. xl;

Ol. cinnamon, gtt, xxx;

Syrup. simplex. ℥ iijss. M.

The patient, if strong or old enough, may gargle with this mixture, or the fauces and pharynx may be swabbed by an assistant; and though this mixture may be found pretty sharp at first, yet the comfort that immediately follows its use makes the patient not only reconciled, but eager to have it used.

**Disease of Stomach.**

Dr. Wilks says that in cases of persistent vomiting, accompanied by hematemesis, it is necessary to ascertain whether the food comes up immediately, or after it has passed into and remained some time in the stomach. In the former case, there may be some contraction of the œsophagus; in the latter there is a probability of the stomach being diseased either with cancer or ulcerated. In cases of gastric ulcer, hematemesis may be slight or severe; when arising from disease of the liver, it is always excessive. He looks with disfavor on the plan of passing bougies for the treatment of œsophageal disease from morbid deposit, since the contraction of the tube from this cause is not at all analogous to that of stricture of the urethra.—*Med. Press & Circ.*

**Constipation.**

The *American Practitioner* says that Prof. Amor has been in the habit of using belladonna in the form of a suppository, in constipation; but following the suggestion of Dr. Baxter, tried the *extract of Stramonium* in the same way, and is much pleased with the result. It posesses, in his judgment, valuable alterative properties, which commends its use in many cases of constipation, independently of its action upon the bowels. Half or three-fourths of a grain of extract of stramonium may be combined with a sufficient quantity of cocoa butter, made into a suppository, and used by the patient each night on going to bed. It is admirably adapted in this form to obstinate constipation of nervous females, who suffer at the same time from pelvic irritations from various causes. It quiets irritation of the uterus and bladder, calms and soothes the nervous system, allays irrritative actions generally, and permits the patient to sleep.

To give permanency, however, to its effects, its use may be accompanied or followed by small doses of nux vomica, or a dinner composed of aloes and nux vomica. Universal and permanent tonic action of the paralyzed muscles of organic life is severed, and the morbid condition of the intestinal glands at the same time corrected.

